# Serum folate, vitamin B-12 and cognitive function in middle and older age: The HAPIEE study

**DOI:** 10.1016/j.exger.2016.01.011

**Published:** 2016-04

**Authors:** Pia Horvat, Julian Gardiner, Ruzena Kubinova, Andrzej Pajak, Abdonas Tamosiunas, Ben Schöttker, Hynek Pikhart, Anne Peasey, Eugene Jansen, Martin Bobak

**Affiliations:** a Department of Epidemiology & Public Health, UCL, 1-19 Torrington Place, London WC1E 7HB, UK; b Department of Education, University of Oxford, 15 Norham Gardens, Oxford OX2 6PY, UK; c National Institute of Public Health, Srobarova 48, 10042 Prague, Czech Republic; d Department of Epidemiology and Population Studies, Jagellonian University Collegium Medicum, Grzegorzecka 20, 31-531 Krakow, Poland; e Department of Population Studies, Institute of Cardiology, Lithuanian University of Health Sciences, Kaunas LT-50161, Lithuania; f Division of Clinical Epidemiology and Aging Research, German Cancer Research Center, Im Neuenheimer Feld 581, 69120 Heidelberg, Germany; g Center for Health Protection, National Institute for Public Health and the Environment, PO Box 1, 3720 BA, Bilthoven, The Netherlands

**Keywords:** Biomarkers, Cognitive function, Ageing, Folate, Vitamin B-12, Eastern Europe

## Abstract

**Background:**

Nutrient status of B vitamins, particularly folate and vitamin B-12, may be related to cognitive ageing but epidemiological evidence remains inconclusive.

**Objective:**

The aim of this study was to estimate the association of serum folate and vitamin B-12 concentrations with cognitive function in middle-aged and older adults from three Central and Eastern European populations.

**Methods:**

Men and women aged 45–69 at baseline participating in the Health, Alcohol and Psychosocial factors in Eastern Europe (HAPIEE) study were recruited in Krakow (Poland), Kaunas (Lithuania) and six urban centres in the Czech Republic. Tests of immediate and delayed recall, verbal fluency and letter search were administered at baseline and repeated in 2006–2008. Serum concentrations of biomarkers at baseline were measured in a sub-sample of participants. Associations of vitamin quartiles with baseline (n = 4166) and follow-up (n = 2739) cognitive domain-specific z-scores were estimated using multiple linear regression.

**Results:**

After adjusting for confounders, folate was positively associated with letter search and vitamin B-12 with word recall in cross-sectional analyses. In prospective analyses, participants in the highest quartile of folate had higher verbal fluency (p < 0.01) and immediate recall (p < 0.05) scores compared to those in the bottom quartile. In addition, participants in the highest quartile of vitamin B-12 had significantly higher verbal fluency scores (β = 0.12; 95% CI = 0.02, 0.21).

**Conclusions:**

Folate and vitamin B-12 were positively associated with performance in some but not all cognitive domains in older Central and Eastern Europeans. These findings do not lend unequivocal support to potential importance of folate and vitamin B-12 status for cognitive function in older age. Long-term longitudinal studies and randomised trials are required before drawing conclusions on the role of these vitamins in cognitive decline.

## Highlights

• Epidemiological evidence linking B vitamins to cognitive ageing is inconclusive.
• Serum folate, vitamin B-12 and cognition were studied in older Eastern Europeans.
• Both vitamins were positively associated with 3-year verbal fluency.
• Folate and vitamin B-12 may be related to performance in some cognitive domains.
• More research is needed to clarify the causal relevance of B vitamins to cognition.

## 1 Introduction

Maintaining cognitive function is a vital part of healthy ageing. With population ageing, understanding the factors which contribute to cognitive function and cognitive decline in older age has gained public health importance. Nutritional factors have been identified as potentially important for cognitive function in later life (Beydoun et al., 2014, Plassman et al., 2010). Folate and vitamin B-12 are among the nutrients with potential significance for retention of cognitive capabilities and prevention of cognitive decline in old age.

The roles of folate and vitamin B-12 in the central nervous system function are well-known and optimal status of these B-vitamins is likely to be important for cognitive function across the life course. Folate is essential for neural tube development in infancy, and both folate and vitamin B-12 are involved in maintaining normal nervous system function at all ages due to their crucial roles in the conversion of homocysteine to methionine (Reynolds, 2006). Suboptimal dietary intakes and/or age-related changes in absorption and metabolism of folate and vitamin B-12 can result in dysregulation of the homocysteine cycle and elevated blood homocysteine levels (i.e. hyper-homocysteinaemia) which may subsequently impair cognition either as an independent risk factor for cardiovascular disease (Wald et al., 2002) or through direct neurotoxic effects (Reynolds, 2006). (Reynolds, 2006). It is theoretically possible that B vitamins affect cognitive ageing through mechanisms other than homocysteine lowering. For example, the folate cycle involves synthesis of methyl groups which are essential for a number of genomic and non-genomic methylation reactions (Reynolds, 2006), including the synthesis of S-adenosylmethionine, the methyl donor for the central nervous system (Morris, 2012), as well as methyl groups used in synthesis of DNA nucleotides. The latter, in particular, could stimulate adult neurogenesis in the hippocampus which is important for memory, sensitive to ageing-related changes (Morris, 2012) and one of the first regions of the brain to show signs of atrophy in Alzheimer's disease. Finally, low vitamin B 12 status may increase cardiovascular risk partly through direct effects, and not exclusively through hyperhomocysteinemia, with potential consequences for cognition; however, the evidence supporting this hypothesis is limited (Rafnsson et al., 2011).

The epidemiological evidence on the associations of blood folate and vitamin B-12 with cognitive ageing remains inconclusive. Numerous studies have reported cross-sectional associations between blood folate and vitamin B-12 and cognitive function (de Lau et al., 2007, Duthie et al., 2002, Elias et al., 2006, Feng et al., 2006, Kado et al., 2005, Ramos et al., 2005, Tucker et al., 2005), although the evidence seems stronger for folate than for vitamin B 12. Evidence from prospective studies is less consistent. Significant associations between low blood folate and vitamin B-12 and poorer cognition or accelerated cognitive decline have also been reported by some (Clarke et al., 2007, Feng et al., 2006, Morris, 2012, Tangney et al., 2009) but not all (de Lau et al., 2009, Kang et al., 2006, Langa et al., 2008, Mooijaart et al., 2005) longitudinal studies.

The homocysteine hypothesis of cardiovascular disease suggests that cognitive domains which are sensitive to vascular risk factors are also vulnerable to low folate and vitamin B 12 status. Folate, vitamin B 12 and homocysteine may also be differentially related to specific cognitive domains; elevated homocysteine has been linked to slower processing speed, low folate to impaired memory function, and low vitamin B-12 to both slower processing speed and worse memory performance but only in studies using sensitive markers of vitamin B 12, such as methylmalonic acid and holotranscobalmin (Morris, 2012). Despite some evidence linking folate and vitamin B-12 to specific cognitive domains, the inconsistency of associations in terms of cognitive tests and domains within and across studies (Doets et al., 2013) makes it difficult to draw firm conclusions about domain-specific associations and the differential contributions, if any, of these B vitamins.

The aim of this study is to examine cross-sectional and prospective associations between serum biomarkers of folate and vitamin B-12 and cognitive function in middle and older age using well-characterised samples from three Central and Eastern European populations.

## 2 Methods

### 2.1 Study populations and participants

The HAPIEE (Health, Alcohol and Psychosocial factors In Eastern Europe) study protocol has been described in detail elsewhere (Peasey et al., 2006). Briefly, random samples of 36,106 men and women aged 45–69 years at baseline were recruited in Novosibirsk (Russia), Krakow (Poland), Kaunas (Lithuania) and six middle-sized urban centres in the Czech Republic using population registers. The baseline survey was conducted in 2002–2005 in the Czech Republic, Krakow and Novosibirsk, and in 2006–2008 in Kaunas which joined the study later. Average response rate was 61%. The baseline survey consisted of an extensive health questionnaire and a brief physical examination, including a venous blood sample. Czech and Krakow participants completed the survey questionnaire during a nurse visit to their home and were subsequently invited to attend a medical examination in a clinic. Novosibirsk and Kaunas participants completed both the questionnaire and the medical examination during a clinic visit. In 2006–2008, Czech, Krakow and Novosibirsk participants were re-interviewed with an average response rate of 63%. In 2002–2005, all participants older than 60 years and a random sample of approximately 20% of younger participants in the Czech Republic, Krakow and Novosibirsk underwent a cognitive assessment. In Krakow, cognitive assessment was conducted in participants' homes; in all other centres it was conducted as part of the clinic visit. In 2006–2008, cognitive assessment was completed for all Kaunas participants and all returning participants in the remaining three centres (n = 17,543); for 54% (n = 9436) of returning participants this was their first assessment of cognitive function.

Fasting venous blood samples were collected during the clinic visits in all centres, following standardized protocols for collection and storage of samples. Due to legal restrictions on exporting blood samples from Russia (n = 9360), biomarkers were measured only in the Czech, Krakow and Kaunas samples (n = 26,746). Biomarkers were analysed in a nested case–control sub-study. The selection of participants in the nested case–control study was as follows: of 26,746 participants, 3462 were excluded because blood samples were unavailable, another 1867 participants withdrew from follow-up, 208 had an unconfirmed cardiovascular event and 3324 had a previous history of cardiovascular disease. Among the 17,885 remaining participants, there were 1882 cases (those who died from any cause or experienced a non-fatal cardiovascular event during follow-up) and 16,003 potential controls. Biomarkers were measured in the 1882 cases and 4476 randomly-selected controls frequency matched to cases by cohort, sex and age group.

The study was approved by ethics committees at University College London and University College Hospital and local ethics committees in each participating centre. Written informed consent was obtained from all participants.

### 2.2 Measurements

#### 2.2.1 Cognitive function

Cognitive function was assessed by four neuropsychological tests (Horvat et al., 2015): i.) immediate word recall (10 nouns over three consecutive 1 min trials); ii.) delayed recall (10 nouns following an interval), both used as tests of verbal memory and learning; iii.) category verbal fluency (animal naming) used as a measure of language and executive function; and iv.) timed letter search used to assess concentration, mental speed and accuracy. Given the previously observed domain-specific associations (Morris, 2012), we might expect both folate and vitamin B 12 to be associated with tests of memory, whereas vitamin B 12 status may also be associated with performance on the timed letter search task. Associations of folate and vitamin B 12 status with category fluency performance have also been reported by some studies (Feng et al., 2006, Hooshmand et al., 2012).

#### 2.2.2 Folate and vitamin B-12

Fasting blood samples were collected in Becton Dickinson SST II (10 ml) and K_2_-EDTA vacutainers (10 ml and 2 × 3 ml). All vacutainers were stored at 4 degrees Celsius prior to processing. The 10 ml SST II and 10 ml K_2_-EDTA vacutainers were centrifuged at 4000 rpm for 15 min, and serum and plasma samples were each divided into several aliquots. All aliquots were stored in 1.5 ml Sarstedt microtubes at − 80 °C for subsequent laboratory analysis. Folate and vitamin B12 were determined in serum with a homogenous chemiluminescent immunoassay using the Access-2 immuno-analyser of Beckman-Coulter, Woerden, the Netherlands. Folate concentrations were expressed in ng/ml, over a detectable range of 0.5 to 20.0. Vitamin B-12 concentrations were expressed in pmol/l, over a detectable range of 37 to 1100. The inter-assay variation was 4.4% for folate and 5.7% for vitamin B-12.

#### 2.2.3 Covariates

In order to minimize potential confounding, covariates were selected based on existing literature (7), hypothesised relationships with the outcomes, and associations with exposures or outcomes in the current cohorts. The following potential confounders were included in the statistical analyses: age at cognitive assessment, sex, study centre, education (primary or less, more than primary but less than tertiary, and tertiary), current economic activity (employed, self-employed, working pensioner, non-working pensioner, unemployed, and other (e.g. housewife, disabled)), smoking status (never, current, former), average alcohol use in last 12 months (0 g/day, < 5/10 g/day, 5–20/10–50 g/day, and > 20/50 g/day in women and men, respectively), and case–control status. We also adjusted for potential mediators: coronary heart disease, stroke, hypertension, and diabetes (all self-reported and dichotomous; yes vs. no).

### 2.3 Statistical analysis

We estimated cross-sectional and prospective associations between B-vitamin biomarkers and cognitive function using four cognitive measures from two study waves (baseline and re-examination) as outcomes and serum folate and vitamin B-12 measured at baseline as the main exposure variables. Participants were eligible for inclusion in the present analysis if they had biomarker measurements at baseline and at least one cognitive function measurement (from baseline and/or re-examination). In cross-sectional analyses, only variables measured at baseline were used; in prospective analyses, we used cognitive function from re-examination and biomarkers and covariates from baseline. Prospective analyses were restricted to participants from Czech towns and Krakow, as only the baseline survey was available in Kaunas. For prospective analysis, follow-up time was defined as the time between measurement of vitamin biomarkers in serum at baseline (2002–05) and measurement of cognitive performance at re-examination (2006–2008).

All cognitive outcomes were converted to z-scores (mean = 0; sd = 1) using the full study sample means and standard deviations to allow comparison between tests (Horvat et al., 2015). Biomarkers were categorized into quartiles (see Table A.1 for cut-off values). For each cognitive test, separate multiple linear regression models were fitted for quartiles of each biomarker. In the test for trend analysis, we modelled the biomarker quartiles as continuous. Additionally, regression models for each cognitive outcome were re-fitted using log-transformed folate or vitamin B-12 concentrations as independent variable. The regression models were initially adjusted only for age and sex, and then additionally adjusted for the remaining confounders and, finally, for potential mediators. All models were adjusted for cohort and case–control status. Models using cognitive measures from re-examination as outcomes were also adjusted for cognitive testing occasion (first test vs. re-test) in order to control for potential confounding by learning effects from repeated test taking. Analyses were conducted in participants with complete data on all model variables. We also tested for possible interactions of biomarkers with sex, age, cohort and alcohol intake.

A number of sensitivity analyses were conducted to examine whether the potential associations were mainly due to biomarkers and/or cognitive outcomes acting as indicators of underlying disease or ill-health and to check the overall robustness of the conclusions. First, we repeated the analysis using only controls because overrepresentation of cases who were generally less healthy and more likely to die or drop-out before re-examination in the nested case–control design could have affected the generalizability of our findings. We also re-estimated the regression models after excluding: 1.) participants who died within 2 years of the baseline survey, 2.) participants with biomarker values at the extremes of the distribution (below 5th or above 95th percentile), 3.) participants who experienced a fatal or non-fatal coronary event or stroke during follow-up, and 4.) participants with prevalent coronary heart disease, stroke or diabetes. Finally, we also re-estimated the regression models after additionally controlling for follow-up time. Analyses were conducted in Stata 13 (StataCorp, 2011).

## 3 Results

Descriptive characteristics of the study sample are shown in Table 1 (and by case–control status in Table A.2). Biomarker and cognitive function data were available for 4512 participants for cross-sectional and 3044 participants for prospective analysis. After excluding observations with any missing values from the analysis, the numbers of participants with complete data were 4166 in cross-sectional and 2739 in prospective analyses. For prospective analysis, mean follow-up time was 3.8 ± 0.4 years (range 1.8–5.5 years). Of the 2739 participants in prospective analysis, 1702 had repeated cognitive measures, and had a mean age of 64.7 years at baseline and 68.4 years at re-examination. Mean age of participants for prospective sample as a whole was 65.3 years, and 64.5 years for the cross-sectional sample. Over 66% of the samples were from men; because of higher male cardiovascular and total mortality men were overrepresented in the nested case–control study. Average baseline values were 8.7 ng/ml (± 4.0) for folate and 243.8 pmol/l (± 115.1) for vitamin B-12.

Results of fully-adjusted multiple regression analyses for folate and cognitive performance are shown in Table 2. In cross-sectional analysis, high concentrations of serum folate were significantly associated with better letter search performance. The associated p-value for trend indicated statistical significance (p < 0.01), as did the p-value (p < 0.01) from analysis with log-transformed folate as the independent variable. Additionally, participants in the third quartile of serum folate had higher verbal fluency scores compared to participants in the bottom quartile. In prospective models, high folate concentrations were also significantly associated with better performance on verbal fluency, and the associated p-value for linear trend (p-value < 0.01) and log-transformed results (p-value = 0.03) were statistically significant. In prospective analysis, the difference between top and bottom quartiles of folate also reached statistical significance for immediate recall but not for letter search. Delayed recall was not associated with serum folate levels in any of the analyses.

Fully-adjusted regression results for vitamin B-12 and cognition are shown in Table 3. Participants in the top quartile of serum vitamin B-12 had significantly better scores on both immediate and delayed recall tests in cross-sectional models, and the associated p-values for trend were significant. These associations were not replicated in prospective models; differences in memory scores between top and bottom quartiles of serum vitamin B-12 were not significant. However, high levels of vitamin B-12 were prospectively associated with better verbal fluency relative to being in the lowest quartile of the biomarker. The associated test for trend and the p-value for the association with log-transformed vitamin B-12 were also significant. The association between serum vitamin B-12 concentrations and letter search was not in the expected direction, although, with the exception of second quartile in cross-sectional analysis, it was not statistically significant.

There were few statistically significant interactions, mostly at 5% level of significance, between biomarkers and age, sex, cohort or alcohol intake in the association with cognitive test performance. It is likely that some borderline significant interactions resulted from multiple testing. Results of sensitivity analysis suggested that the observed associations, or lack thereof, between both vitamin biomarkers and cognitive outcomes were generally robust (reported in Tables A.3–A.7). After excluding cases from the analysis, cross-sectional and prospective associations between the vitamins and verbal fluency were slightly strengthened (Table A.3). However, after excluding participants with pre-existing cardiovascular disease and diabetes from the analysis, the associations between the vitamins and verbal fluency were slightly attenuated (Table A.7).

## 4 Discussion

This study found evidence of positive associations between folate and vitamin B-12 concentrations and performance in some but not all cognitive domains in a cohort of middle-aged and older Central and Eastern Europeans. Folate was positively associated with verbal fluency and borderline associated with immediate recall in prospective analyses. Furthermore, higher concentrations of folate were associated with better letter search performance in cross-sectional analysis. Associations between vitamin B-12 and memory, observed cross-sectionally, were not detected in prospective analysis but high levels of vitamin B-12 were prospectively associated with better verbal fluency. This association followed a significant linear trend.

The results of our study should be interpreted in the context of its limitations. First, the analytic sample was restricted to participants with complete data. Although biomarkers were only measured in a case–control subsample, inclusion of the variables used in the selection of the case–control subsample (sex, age, cohort and case–control status) as covariates in the regression models is considered to result in unbiased estimates using complete case analysis in absence of inverse probability weighting. However, missing data on covariates and cognitive function could still have affected our results, in particular longitudinal attrition in prospective analyses.

Second, response rates were relatively low, although this is typical for most contemporary epidemiologic studies (Galea and Tracy, 2007). As responders tended to be healthier than non-responders (Peasey et al., 2006), participation rates may have been lower in those with poor cognition and unfavourable vitamin profiles. Because of this, it is possible that the absolute levels of cognitive performance and biomarkers were overestimated in our study. However, nonresponse rates are not a good predictor of nonresponse bias (Groves, 2006), and effect estimates of associations between vitamin biomarkers and cognitive function may be relatively unbiased.

Third, we did not have direct information on dietary supplementation of folate and vitamin B-12. It is possible that those who take dietary supplements have healthier behaviours which may positively impact cognitive performance. However, the prevalence of any vitamin supplementation is low in the HAPIEE cohorts, with no clear inverse association between antioxidant vitamin intake and subsequent mortality (Stepaniak et al., 2015). In addition, our analytical models were adjusted for the main health behaviours, including smoking and alcohol consumption.

While not a direct limitation, we have not measured blood homocysteine and could not explore its association with folate and vitamin B-12 status in the relationship with cognitive function. It would also be interesting to compare mean values for serum folate and vitamin B 12 status and mean cognitive performance in Central and Eastern European cohorts with other European or international cohorts. However, methodological differences in measurement of biomarkers and cognition between our study and previous studies (Bobak et al., 2009) make such direct comparisons difficult.

Strengths of this study include objective assessments of vitamin biomarkers and cognitive function, and relatively large well-characterised samples from three Central and Eastern European populations.

In our study, folate was positively associated with verbal fluency. We also observed a weakly significant prospective association between high folate levels and better immediate recall, and better performance on the letter search task, assessing mental speed, in cross-sectional analysis. Most cross-sectional studies of blood folate and performance on neuropsychological tests found a significant association with at least one test, although studies of memory were almost as likely to find no association as a significant association (Morris, 2012). However, cross-sectional studies cannot establish a temporal relationship between the exposure and the outcome.

Our observation that folate was prospectively associated with verbal fluency (and weakly associated with working memory) over a 3-year follow-up period, raises the possibility of a causal association, at least in some cognitive domains. A previous longitudinal study linked high serum folate to slower decline in spatial copying ability and high dietary folate with improved verbal fluency over a 3-year follow-up period (Tucker et al., 2005). In another prospective study, low folate status was linked to accelerated 7-year decline in global cognition (Kado et al., 2005). However, not all prospective studies have observed an association (Clarke et al., 2007, Kang et al., 2006). Results from randomised trials have been similar. Folic acid supplementation was associated with improved domain-specific cognitive performance in two (Durga et al., 2007, Walker et al., 2012) of the three randomised trials with relatively large samples and two or more years of follow-up (Durga et al., 2007, McMahon et al., 2006, Walker et al., 2012).

We observed cross-sectional but not prospective associations between vitamin B-12 and memory. Vitamin B-12 was prospectively associated with better verbal fluency, and the association followed a linear trend. Our results do not suggest a particularly strong relationship between vitamin B-12 and cognitive performance in middle and older age. This is broadly in line with a recent systematic review and meta-analysis of prospective studies which did not find consistent evidence for the hypothesis relating vitamin B-12 status to global or domain-specific cognitive performance in older people (Doets et al., 2013). Similar to our study, some prospective cohort studies have reported positive domain-specific findings, particularly between vitamin B-12 and executive function or memory, but the results have been inconsistent across studies (Doets et al., 2013). In a recent randomised trial, vitamin B-12 supplementation was not associated with improved cognitive and neurological functions after 12 months in 201 participants with mild vitamin B-12 deficiency (Dangour et al., 2015). These findings do not support the hypothesis that optimizing vitamin B-12 status benefits cognitive function in older age, although with a mean age of 80 years the results of this trial are not directly comparable to our study population.

It is also possible that serum vitamin B-12 is not sensitive enough to detect associations with cognitive ageing. Other markers, such as methylmalonic acid and holotranscobalamin, are thought to be more sensitive but are not yet routinely measured in population studies. Studies which used these markers were more likely to observe positive associations between low vitamin B-12 concentrations and increased cognitive risk but such studies are currently few in number (Clarke et al., 2007; Doets and others; Morris et al., 2012, O'Leary et al., 2012).

There is no universally agreed measurement standard for determination of folate and vitamin B-12 status, and the interpretation of lower values in not always clear. In addition, variation in blood folate and vitamin B-12 levels within the currently considered normal range may also be significant for cognitive function. We explored a dose–response relationship between folate and vitamin B-12 and cognitive performance, and found evidence for a significant linear trend in prospective associations of both folate and vitamin B-12 with verbal fluency, as well as cross-sectional associations between folate and timed letter search and vitamin B-12 and memory.

## 5 Conclusions

Folate and, to a lesser extent, vitamin B-12 were positively associated with performance in some cognitive domains in our study in middle-aged and older Central and Eastern Europeans. Both folate and vitamin B-12 were prospectively associated with verbal fluency over a 3-year follow-up period. Since prospective associations are less likely to be affected by reverse causation, this finding is, at least, suggestive of a possible association in specific cognitive domains. However, neither vitamin showed consistent relationships both cross-sectionally and prospectively or across cognitive tests. Longitudinal studies with longer follow-ups, high-quality randomised trials and genetic studies are needed before drawing firm conclusions on the causal role of folate and vitamin B-12 in cognitive ageing and potential preventative or therapeutic benefits for cognition of optimizing folate and vitamin B-12 status in older people.

## Figures and Tables

**Table 1:** Descriptive characteristics of the study sample.

	Cross-sectional (n = 4166)		Prospective (n = 2739)	
	Mean/ n	(SD)/ (%)	Mean/ n	(SD)/ (%)
Folate (ng/ml)	8.7	(4.0)	8.7	(4.0)
Vitamin B-12 (pmol/l)	243.8	(115.1)	246.7	(112.1)
Age	64.5	(5.4)	65.3	(6.4)
Immediate recall	20.5	(4.1)	21.4	(4.1)
Delayed recall	7.1	(1.9)	7.1	(1.9)
Verbal fluency	20.8	(6.4)	22.5	(6.4)
Letter search	16.3	(5.2)	17.3	(4.7)

*Center*				
Czech towns	1355	32.5	1403	51.2
Krakow (Poland)	1163	27.9	1336	48.8
Kaunas (Lithuania)	1648	39.6	NA	NA

*Sex*				
Male	2768	66.4	1844	67.3

Education				
Primary or less	498	12.0	244	8.9
Secondary	2314	55.5	1804	65.9
College or university	1354	32.5	691	25.2

*Current economic activity*				
Full-time/part-time employed	609	14.6	605	22.1
Self-employed	87	2.1	138	5.0
Pensionable age, still working	641	15.4	300	11.0
Pensionable age, not working	2689	64.5	1607	58.7
Unemployed	54	1.3	57	2.1
Other	86	2.1	32	1.2

*Smoking status*				
Never smoker	1932	46.4	1111	40.6
Former smoker	1295	31.1	934	34.1
Current smoker	939	22.5	694	25.3

*Self-reported history of*				
Myocardial infarction	461	11.1	227	8.3
Stroke	176	4.2	81	3.0
Hypertension	2520	60.5	1485	54.2
Diabetes	591	14.2	381	13.9

*Alcohol intake (g/day)*				
0	722	17.3	539	19.7
< 5/10	2473	59.4	1512	55.2
5–20/10–50	819	19.7	535	19.5
> 20/50	152	3.6	153	5.6

*Case–control status*				
Case	1195	28.7	475	17.3
Control	2971	71.3	2264	82.7

**Table 2:** Linear regression results for associations of serum folate (ng/ml) with standardized cognitive scores.

	Immediate recall		Delayed recall		Verbal fluency		Letter search	
	b	95% CI	b	95% CI	b	95% CI	b	95% CI
Cross-sectional (n = 4166)^a^								
Folate (ng/ml)								
1st quartile	0.00	[0.00, 0.00]	0.00	[0.00, 0.00]	0.00	[0.00, 0.00]	0.00	[0.00, 0.00]
2nd quartile	0.03	[− 0.05, 0.10]	0.05	[− 0.03, 0.13]	0.02	[− 0.05, 0.09]	0.03	[− 0.05, 0.11]
3rd quartile	0.05	[− 0.03, 0.12]	0.05	[− 0.03, 0.13]	0.07⁎	[0.00, 0.15]	0.11⁎⁎	[0.03, 0.20]
4th quartile	0.06	[− 0.01, 0.14]	0.04	[− 0.04, 0.12]	0.05	[− 0.02, 0.13]	0.10⁎	[0.01, 0.18]
p for trend		0.083		0.362		0.070		0.008
p for logged folate^c^		0.111		0.055		0.132		0.007

Prospective (n = 2739)^b^								
Folate (ng/ml)								
1st quartile	0.00	[0.00, 0.00]	0.00	[0.00, 0.00]	0.00	[0.00, 0.00]	0.00	[0.00, 0.00]
2nd quartile	0.05	[− 0.04, 0.15]	0.01	[− 0.09, 0.11]	0.05	[− 0.04, 0.14]	0.01	[− 0.09, 0.11]
3rd quartile	0.06	[− 0.03, 0.16]	− 0.01	[− 0.11, 0.08]	0.09	[− 0.01, 0.18]	− 0.01	[− 0.10, 0.09]
4th quartile	0.10⁎	[0.00, 0.20]	0.02	[− 0.08, 0.12]	0.13⁎⁎	[0.03, 0.22]	0.07	[− 0.03, 0.16]
p for trend		0.050		0.839		0.006		0.251
p for logged folate		0.077		0.306		0.031		0.207

⁎ p ≤ 0.05.

⁎⁎ p ≤ 0.01.

a Cross-sectional models were adjusted for age, sex, study centre, education, current economic activity, smoking, alcohol, self-reported history of chronic conditions and case–control status.

b Prospective models were additionally adjusted for cognitive testing occasion.

c p-Value associated with log-transformed biomarker used as independent variable in regression analysis.

**Table 3:** Linear regression results for associations of serum vitamin B-12 (pmol/l) with standardized cognitive scores.

	Immediate recall		Delayed recall		Verbal fluency		Letter search	
	b	95% CI	b	95% CI	b	95% CI	b	95% CI
Cross-sectional (n = 4166)^a^								
Vitamin B-12 (pmol/l)								
1st quartile	0.00	[0.00, 0.00]	0.00	[0.00, 0.00]	0.00	[0.00, 0.00]	0.00	[0.00, 0.00]
2nd quartile	0.04	[− 0.04, 0.11]	0.06	[− 0.01, 0.14]	− 0.02	[− 0.09, 0.05]	− 0.09⁎	[− 0.18,-0.01]
3rd quartile	0.05	[− 0.02, 0.13]	0.05	[− 0.03, 0.13]	− 0.00	[− 0.07, 0.07]	− 0.06	[− 0.14, 0.03]
4th quartile	0.09⁎	[0.02, 0.17]	0.09⁎	[0.01, 0.17]	0.05	[− 0.02, 0.12]	− 0.08	[− 0.17, 0.00]
p for trend		0.016		0.038		0.137		0.115
p for logged vitamin B-12^c^		0.007		0.022		0.106		0.048

Prospective (n = 2739)^b^								
Vitamin B-12 (pmol/l)								
1st quartile	0.00	[0.00, 0.00]	0.00	[0.00, 0.00]	0.00	[0.00, 0.00]	0.00	[0.00, 0.00]
2nd quartile	0.00	[− 0.09, 0.10]	− 0.00	[− 0.10, 0.10]	0.01	[− 0.08, 0.10]	− 0.02	[− 0.12, 0.07]
3rd quartile	0.08	[− 0.02, 0.17]	0.08	[− 0.02, 0.18]	0.06	[− 0.03, 0.16]	0.06	[− 0.04, 0.15]
4th quartile	0.06	[− 0.04, 0.16]	0.04	[− 0.06, 0.14]	0.12⁎	[0.02, 0.21]	− 0.00	[− 0.10, 0.10]
p for trend		0.098		0.190		0.007		0.590
p for logged vitamin B-12		0.043		0.203		0.024		0.798

⁎⁎ p ≤ 0.01.

⁎ p ≤ 0.05.

a Cross-sectional models were adjusted for age, sex, study centre, education, current economic activity, smoking, alcohol, self-reported history of chronic conditions and case–control status.

b Prospective models were additionally adjusted for cognitive testing occasion.

c p-Value associated with log-transformed biomarker used as independent variable in regression analysis.
