# Supplementary material for: Serum folate, vitamin B-12 and cognitive function in middle and older age: The HAPIEE study
Source: Exp Gerontol. 2016 Apr;76:33–8. doi: 10.1016/j.exger.2016.01.011 (PMC4839985; doi:10.1016/j.exger.2016.01.011)
Supplement: Supplementary file 1 — Supplementary material. [file mmc1.docx]

**Serum folate, vitamin B-12 and cognitive function in middle and older age: the HAPIEE study**

Supplementary appendix

Contents

[Table A.1 Cut-off values for vitamin biomarker quartiles 2](#_Toc432420326)

[Table A.2 Descriptive characteristics of the study sample by case control status 3](#_Toc432420327)

[Table A.3 Linear regression results for associations of serum vitamins with standardized cognitive scores in controls only (cases were excluded) 4](#_Toc432420328)

[Table A.4 Linear regression results for associations of serum vitamins with standardized cognitive scores excluding participants who died within 2 years from the baseline examination 5](#_Toc432420329)

[Table A.5 Linear regression results for associations of serum vitamins with standardized cognitive scores excluding participants with fatal or non-fatal coronary event or stroke at follow-up 6](#_Toc432420330)

[Table A.6 Linear regression results for associations of serum vitamins with standardized cognitive scores excluding participants with extreme biomarker values 7](#_Toc432420331)

[Table A.7 Linear regression results for associations of serum vitamins with standardized cognitive scores excluding observations with pre-existing CVD or diabetes 8](#_Toc432420332)

# Table A.1 Cut-off values for vitamin biomarker quartiles

|  | **Folate**  **(ng/mL)** |  | **Vitamin B-12**  **(pmol/L)** |
| --- | --- | --- | --- |
| Cut-off values |  |  |  |
| 1^st^ quartile | 5.8 |  | 171.0 |
| 2^nd^ quartile | 7.7 |  | 224.0 |
| 3^rd^ quartile | 10.5 |  | 292.0 |
| 4^th^ quartile | 20 |  | 1100.0 |

# Table A.2 Descriptive characteristics of the study sample by case control status

|  | **Cross-sectional** | | | |  |  |  | **Prospective** | | | |
| --- | --- | --- | --- | --- | --- | --- | --- | --- | --- | --- | --- |
|  | **Control**  **(n=2,971)** | | **Case**  **(n=1,195)** | |  |  |  | **Control**  **(n=2,264)** | | **Case**  **(n=475)** | |
|  | **Mean/**  **n** | **(SD)/**  **(%)** | **Mean/**  **n** | **(SD)/**  **(%)** |  |  |  | **Mean/**  **n** | **(SD)/**  **(%)** | **Mean/**  **n** | **(SD)/**  **(%)** |
| Folate (ng/mL) | 8.8 | (4.0) | 8.2 | (3.9) |  |  |  | 8.8 | (4.0) | 8.2 | (4.0) |
| Vitamin B-12 (pmol/L) | 244.7 | (112.1) | 241.7 | (122.2) |  |  |  | 248.4 | (111.1) | 238.9 | (116.3) |
| Age | 64.5 | (5.4) | 64.5 | (5.4) |  |  |  | 65.4 | (6.4) | 65.0 | (6.4) |
| Immediate recall | 20.8 | (4.0) | 19.8 | (4.3) |  |  |  | 21.5 | (4.0) | 20.8 | (4.3) |
| Delayed recall | 7.2 | (1.8) | 6.8 | (2.0) |  |  |  | 7.2 | (1.9) | 7.0 | (2.0) |
| Verbal fluency | 21.2 | (6.4) | 19.9 | (6.4) |  |  |  | 22.7 | (6.4) | 21.7 | (6.6) |
| Letter search | 16.7 | (5.0) | 15.4 | (5.5) |  |  |  | 17.5 | (4.6) | 16.4 | (4.8) |
| Center |  |  |  |  |  |  |  |  |  |  |  |
| Czech towns | 979 | 33.0 | 376 | 31.5 |  |  |  | 1141 | 50.4 | 262 | 55.2 |
| Krakow (Poland) | 821 | 27.6 | 342 | 28.6 |  |  |  | 1123 | 49.6 | 213 | 44.8 |
| Kaunas (Lithuania) | 1171 | 39.4 | 477 | 39.9 |  |  |  | NA | NA | NA | NA |
| Sex |  |  |  |  |  |  |  |  |  |  |  |
| Male | 1982 | 66.7 | 786 | 65.8 |  |  |  | 1531 | 67.6 | 313 | 65.9 |
| Education |  |  |  |  |  |  |  |  |  |  |  |
| Primary or less | 310 | 10.4 | 188 | 15.7 |  |  |  | 197 | 8.7 | 47 | 9.9 |
| Secondary | 1625 | 54.7 | 689 | 57.7 |  |  |  | 1466 | 64.8 | 338 | 71.2 |
| College or university | 1036 | 34.9 | 318 | 26.6 |  |  |  | 601 | 26.5 | 90 | 18.9 |
| Current economic activity |  |  |  |  |  |  |  |  |  |  |  |
| Full-time/part-time employed | 463 | 15.6 | 146 | 12.2 |  |  |  | 503 | 22.2 | 102 | 21.5 |
| Self-employed | 67 | 2.3 | 20 | 1.7 |  |  |  | 122 | 5.4 | 16 | 3.4 |
| Pensionable age, still working | 509 | 17.1 | 132 | 11.0 |  |  |  | 262 | 11.6 | 38 | 8.0 |
| Pensionable age, not working | 1851 | 62.3 | 838 | 70.1 |  |  |  | 1307 | 57.7 | 300 | 63.2 |
| Unemployed | 29 | 1.0 | 25 | 2.1 |  |  |  | 42 | 1.9 | 15 | 3.2 |
| Other | 52 | 1.8 | 34 | 2.8 |  |  |  | 28 | 1.2 | 4 | 0.8 |
| Smoking status |  |  |  |  |  |  |  |  |  |  |  |
| Never smoker | 1490 | 50.2 | 442 | 37.0 |  |  |  | 954 | 42.1 | 157 | 33.1 |
| Former smoker | 917 | 30.9 | 378 | 31.6 |  |  |  | 787 | 34.8 | 147 | 30.9 |
| Current smoker | 564 | 19.0 | 375 | 31.4 |  |  |  | 523 | 23.1 | 171 | 36.0 |
| Self-reported history of |  |  |  |  |  |  |  |  |  |  |  |
| Myocardial infarction | 272 | 9.2 | 189 | 15.8 |  |  |  | 183 | 8.1 | 44 | 9.3 |
| Stroke | 113 | 3.8 | 63 | 5.3 |  |  |  | 67 | 3.0 | 14 | 2.9 |
| Hypertension | 1759 | 59.2 | 761 | 63.7 |  |  |  | 1193 | 52.7 | 292 | 61.5 |
| Diabetes | 351 | 11.8 | 240 | 20.1 |  |  |  | 288 | 12.7 | 93 | 19.6 |
| Alcohol intake (g/day) |  |  |  |  |  |  |  |  |  |  |  |
| 0 | 463 | 15.6 | 259 | 21.7 |  |  |  | 439 | 19.4 | 100 | 21.1 |
| <5/10 | 1787 | 60.1 | 686 | 57.4 |  |  |  | 1244 | 54.9 | 268 | 56.4 |
| 5-20/10-50 | 620 | 20.9 | 199 | 16.7 |  |  |  | 461 | 20.4 | 74 | 15.6 |
| >20/50 | 101 | 3.4 | 51 | 4.3 |  |  |  | 120 | 5.3 | 33 | 6.9 |
|  |  |  |  |  |  |  |  |  |  |  |  |

# Table A.3 Linear regression results for associations of serum vitamins with standardized cognitive scores in controls only (cases were excluded)

|  | **Immediate**  **recall** | | **Delayed**  **recall** | | **Verbal**  **fluency** | | **Letter**  **search** | |
| --- | --- | --- | --- | --- | --- | --- | --- | --- |
|  | **b** | **95% CI** | **b** | **95% CI** | **b** | **95% CI** | **b** | **95% CI** |
| **Cross-sectional (n=2,971)** |  |  |  |  |  |  |  |  |
| **Folate (ng/mL)** |  |  |  |  |  |  |  |  |
| 1st quartile | 0.00 | [0.00,0.00] | 0.00 | [0.00,0.00] | 0.00 | [0.00,0.00] | 0.00 | [0.00,0.00] |
| 2nd quartile | 0.02 | [-0.07,0.11] | 0.05 | [-0.04,0.15] | 0.03 | [-0.06,0.11] | 0.01 | [-0.09,0.11] |
| 3rd quartile | 0.05 | [-0.04,0.14] | 0.04 | [-0.05,0.14] | 0.09^*^ | [0.00,0.18] | 0.13^*^ | [0.03,0.23] |
| 4th quartile | 0.02 | [-0.07,0.11] | 0.03 | [-0.07,0.12] | 0.06 | [-0.03,0.14] | 0.06 | [-0.04,0.16] |
|  |  |  |  |  |  |  |  |  |
| P for trend |  | (0.505) |  | (0.710) |  | (0.105) |  | (0.070) |
| P for logged |  | (0.475) |  | (0.209) |  | (0.276) |  | (0.074) |
|  |  |  |  |  |  |  |  |  |
| **Prospective (n=2,264)** |  |  |  |  |  |  |  |  |
| **Folate (ng/mL)** |  |  |  |  |  |  |  |  |
| 1st quartile | 0.00 | [0.00,0.00] | 0.00 | [0.00,0.00] | 0.00 | [0.00,0.00] | 0.00 | [0.00,0.00] |
| 2nd quartile | 0.04 | [-0.06,0.15] | -0.02 | [-0.13,0.09] | 0.04 | [-0.07,0.14] | 0.03 | [-0.07,0.14] |
| 3rd quartile | 0.08 | [-0.03,0.18] | -0.03 | [-0.14,0.08] | 0.10 | [-0.01,0.20] | 0.03 | [-0.08,0.13] |
| 4th quartile | 0.08 | [-0.03,0.18] | 0.00 | [-0.11,0.11] | 0.14^**^ | [0.04,0.24] | 0.09 | [-0.02,0.20] |
|  |  |  |  |  |  |  |  |  |
| P for trend |  | (0.125) |  | (0.999) |  | (0.003) |  | (0.125) |
| P for logged |  | (0.173) |  | (0.544) |  | (0.028) |  | (0.097) |
|  |  |  |  |  |  |  |  |  |
| **Cross-sectional (n=2,971)** |  |  |  |  |  |  |  |  |
| **Vitamin B-12 (pmol/L)** |  |  |  |  |  |  |  |  |
| 1st quartile | 0.00 | [0.00,0.00] | 0.00 | [0.00,0.00] | 0.00 | [0.00,0.00] | 0.00 | [0.00,0.00] |
| 2nd quartile | 0.05 | [-0.03,0.14] | 0.06 | [-0.03,0.15] | 0.02 | [-0.06,0.11] | -0.08 | [-0.18,0.01] |
| 3rd quartile | 0.04 | [-0.04,0.13] | 0.04 | [-0.05,0.13] | 0.04 | [-0.05,0.13] | -0.06 | [-0.16,0.04] |
| 4th quartile | 0.10^*^ | [0.01,0.19] | 0.09 | [-0.01,0.18] | 0.09^*^ | [0.00,0.17] | -0.09 | [-0.18,0.01] |
|  |  |  |  |  |  |  |  |  |
| P for trend |  | (0.046) |  | (0.117) |  | (0.041) |  | (0.147) |
| P for logged |  | (0.015) |  | (0.150) |  | (0.058) |  | (0.054) |
|  |  |  |  |  |  |  |  |  |
| **Prospective (n=2,264)** |  |  |  |  |  |  |  |  |
| **Vitamin B-12 (pmol/L)** |  |  |  |  |  |  |  |  |
| 1st quartile | 0.00 | [0.00,0.00] | 0.00 | [0.00,0.00] | 0.00 | [0.00,0.00] | 0.00 | [0.00,0.00] |
| 2nd quartile | -0.01 | [-0.11,0.10] | 0.02 | [-0.09,0.12] | 0.05 | [-0.06,0.15] | 0.01 | [-0.10,0.11] |
| 3rd quartile | 0.05 | [-0.06,0.15] | 0.07 | [-0.04,0.18] | 0.07 | [-0.03,0.17] | 0.11^*^ | [0.01,0.22] |
| 4th quartile | 0.05 | [-0.05,0.16] | 0.05 | [-0.06,0.16] | 0.15^**^ | [0.05,0.25] | 0.03 | [-0.07,0.14] |
|  |  |  |  |  |  |  |  |  |
| P for trend |  | (0.188) |  | (0.217) |  | (0.003) |  | (0.225) |
| P for logged |  | (0.041) |  | (0.132) |  | (0.007) |  | (0.499) |

^*^ P ≤ 0.05, ^**^ P ≤ 0.01, ^***^ P ≤ 0.001

Cross-sectional models were adjusted for age, sex, study centre, education, current economic activity, smoking, alcohol, self-reported history of chronic conditions and case-control status.

Prospective models were additionally adjusted for cognitive testing occasion.

P for trend denotes P-value associated with biomarker quartiles modelled as continuous in regression analysis.

P for logged denotes P-value associated with log-transformed biomarker used as independent variable in regression analysis.

# Table A.4 Linear regression results for associations of serum vitamins with standardized cognitive scores excluding participants who died within 2 years from the baseline examination

|  | **Immediate**  **recall** | | **Delayed**  **recall** | | **Verbal**  **fluency** | | **Letter**  **search** | |
| --- | --- | --- | --- | --- | --- | --- | --- | --- |
|  | **b** | **95% CI** | **b** | **95% CI** | **b** | **95% CI** | **b** | **95% CI** |
| **Cross-sectional (n=3,930)** |  |  |  |  |  |  |  |  |
| **Folate** |  |  |  |  |  |  |  |  |
| 1st quartile | 0.00 | [0.00,0.00] | 0.00 | [0.00,0.00] | 0.00 | [0.00,0.00] | 0.00 | [0.00,0.00] |
| 2nd quartile | 0.03 | [-0.05,0.11] | 0.05 | [-0.03,0.13] | 0.02 | [-0.06,0.09] | 0.04 | [-0.05,0.12] |
| 3rd quartile | 0.05 | [-0.03,0.12] | 0.03 | [-0.05,0.11] | 0.07 | [-0.01,0.14] | 0.11^*^ | [0.02,0.20] |
| 4th quartile | 0.06 | [-0.01,0.14] | 0.04 | [-0.04,0.12] | 0.05 | [-0.02,0.13] | 0.10^*^ | [0.01,0.18] |
|  |  |  |  |  |  |  |  |  |
| P for trend |  | (0.102) |  | (0.459) |  | (0.092) |  | (0.012) |
| P for logged |  | (0.164) |  | (0.084) |  | (0.160) |  | (0.012) |
|  |  |  |  |  |  |  |  |  |
| **Prospective (n=2,739)** |  |  |  |  |  |  |  |  |
| **Folate** |  |  |  |  |  |  |  |  |
| 1st quartile | 0.00 | [0.00,0.00] | 0.00 | [0.00,0.00] | 0.00 | [0.00,0.00] | 0.00 | [0.00,0.00] |
| 2nd quartile | 0.05 | [-0.04,0.15] | 0.01 | [-0.09,0.11] | 0.05 | [-0.04,0.14] | 0.01 | [-0.09,0.11] |
| 3rd quartile | 0.06 | [-0.03,0.16] | -0.01 | [-0.11,0.08] | 0.09 | [-0.01,0.18] | -0.01 | [-0.10,0.09] |
| 4th quartile | 0.10^*^ | [0.00,0.20] | 0.02 | [-0.08,0.12] | 0.13^**^ | [0.03,0.22] | 0.07 | [-0.03,0.16] |
|  |  |  |  |  |  |  |  |  |
| P for trend |  | (0.050) |  | (0.839) |  | (0.006) |  | (0.251) |
| P for logged |  | (0.077) |  | (0.306) |  | (0.031) |  | (0.207) |
|  |  |  |  |  |  |  |  |  |
| **Cross-sectional (n=3,930)** |  |  |  |  |  |  |  |  |
| **Vitamin B-12 (pmol/L)** |  |  |  |  |  |  |  |  |
| 1st quartile | 0.00 | [0.00,0.00] | 0.00 | [0.00,0.00] | 0.00 | [0.00,0.00] | 0.00 | [0.00,0.00] |
| 2nd quartile | 0.04 | [-0.04,0.11] | 0.06 | [-0.02,0.14] | -0.01 | [-0.08,0.07] | -0.09^*^ | [-0.18,-0.01] |
| 3rd quartile | 0.04 | [-0.03,0.12] | 0.05 | [-0.04,0.13] | 0.01 | [-0.06,0.09] | -0.06 | [-0.15,0.03] |
| 4th quartile | 0.09^*^ | [0.02,0.17] | 0.09^*^ | [0.00,0.17] | 0.06 | [-0.01,0.14] | -0.09 | [-0.17,0.00] |
|  |  |  |  |  |  |  |  |  |
| P for trend |  | (0.021) |  | (0.061) |  | (0.100) |  | (0.106) |
| P for logged |  | (0.009) |  | (0.053) |  | (0.083) |  | (0.050) |
|  |  |  |  |  |  |  |  |  |
| **Prospective (n=2,739)** |  |  |  |  |  |  |  |  |
| **Vitamin B-12 (pmol/L)** |  |  |  |  |  |  |  |  |
| 1st quartile | 0.00 | [0.00,0.00] | 0.00 | [0.00,0.00] | 0.00 | [0.00,0.00] | 0.00 | [0.00,0.00] |
| 2nd quartile | 0.00 | [-0.09,0.10] | -0.00 | [-0.10,0.10] | 0.01 | [-0.08,0.10] | -0.02 | [-0.12,0.07] |
| 3rd quartile | 0.08 | [-0.02,0.17] | 0.08 | [-0.02,0.18] | 0.06 | [-0.03,0.16] | 0.06 | [-0.04,0.15] |
| 4th quartile | 0.06 | [-0.04,0.16] | 0.04 | [-0.06,0.14] | 0.12^*^ | [0.02,0.21] | -0.00 | [-0.10,0.10] |
|  |  |  |  |  |  |  |  |  |
| P for trend |  | (0.098) |  | (0.190) |  | (0.007) |  | (0.590) |
| P for logged |  | (0.043) |  | (0.203) |  | (0.024) |  | (0.798) |

^*^ P ≤ 0.05, ^**^ P ≤ 0.01, ^***^ P ≤ 0.001

Cross-sectional models were adjusted for age, sex, study centre, education, current economic activity, smoking, alcohol, self-reported history of chronic conditions and case-control status.

Prospective models were additionally adjusted for cognitive testing occasion.

P for trend denotes P-value associated with biomarker quartiles modelled as continuous in regression analysis.

P for logged denotes P-value associated with log-transformed biomarker used as independent variable in regression analysis.

# Table A.5 Linear regression results for associations of serum vitamins with standardized cognitive scores excluding participants with fatal or non-fatal coronary event or stroke at follow-up

|  | **Immediate**  **recall** | | **Delayed**  **recall** | | **Verbal**  **fluency** | | **Letter**  **search** | |
| --- | --- | --- | --- | --- | --- | --- | --- | --- |
|  | **b** | **95% CI** | **b** | **95% CI** | **b** | **95% CI** | **b** | **95% CI** |
| **Cross-sectional (n=3,639)** |  |  |  |  |  |  |  |  |
| **Folate (ng/mL)** |  |  |  |  |  |  |  |  |
| 1st quartile | 0.00 | [0.00,0.00] | 0.00 | [0.00,0.00] | 0.00 | [0.00,0.00] | 0.00 | [0.00,0.00] |
| 2nd quartile | 0.03 | [-0.05,0.11] | 0.07 | [-0.02,0.15] | 0.04 | [-0.04,0.12] | 0.03 | [-0.06,0.12] |
| 3rd quartile | 0.03 | [-0.05,0.11] | 0.05 | [-0.03,0.14] | 0.08^*^ | [0.01,0.16] | 0.10^*^ | [0.01,0.19] |
| 4th quartile | 0.06 | [-0.02,0.14] | 0.05 | [-0.03,0.14] | 0.07 | [-0.01,0.15] | 0.08 | [-0.01,0.17] |
|  |  |  |  |  |  |  |  |  |
| P for trend |  | (0.190) |  | (0.330) |  | (0.054) |  | (0.036) |
| P for logged |  | (0.212) |  | (0.078) |  | (0.140) |  | (0.028) |
|  |  |  |  |  |  |  |  |  |
| **Prospective (n=2,453)** |  |  |  |  |  |  |  |  |
| **Folate (ng/mL)** |  |  |  |  |  |  |  |  |
| 1st quartile | 0.00 | [0.00,0.00] | 0.00 | [0.00,0.00] | 0.00 | [0.00,0.00] | 0.00 | [0.00,0.00] |
| 2nd quartile | 0.07 | [-0.04,0.17] | 0.00 | [-0.10,0.11] | 0.06 | [-0.04,0.15] | 0.03 | [-0.07,0.13] |
| 3rd quartile | 0.09 | [-0.01,0.19] | -0.01 | [-0.11,0.09] | 0.11^*^ | [0.01,0.21] | 0.02 | [-0.08,0.12] |
| 4th quartile | 0.09 | [-0.01,0.20] | 0.00 | [-0.10,0.11] | 0.15^**^ | [0.05,0.24] | 0.07 | [-0.03,0.17] |
|  |  |  |  |  |  |  |  |  |
| P for trend |  | (0.065) |  | (0.992) |  | (0.002) |  | (0.224) |
| P for logged |  | (0.147) |  | (0.681) |  | (0.014) |  | (0.237) |
|  |  |  |  |  |  |  |  |  |
| **Cross-sectional (n=3,639)** |  |  |  |  |  |  |  |  |
| **Vitamin B-12 (pmol/L)** |  |  |  |  |  |  |  |  |
| 1st quartile | 0.00 | [0.00,0.00] | 0.00 | [0.00,0.00] | 0.00 | [0.00,0.00] | 0.00 | [0.00,0.00] |
| 2nd quartile | 0.04 | [-0.04,0.12] | 0.06 | [-0.02,0.15] | -0.02 | [-0.10,0.05] | -0.09 | [-0.17,0.00] |
| 3rd quartile | 0.07 | [-0.01,0.15] | 0.06 | [-0.03,0.14] | 0.02 | [-0.06,0.10] | -0.04 | [-0.13,0.05] |
| 4th quartile | 0.08^*^ | [0.00,0.16] | 0.08 | [-0.00,0.17] | 0.04 | [-0.03,0.12] | -0.08 | [-0.17,0.01] |
|  |  |  |  |  |  |  |  |  |
| P for trend |  | (0.030) |  | (0.076) |  | (0.152) |  | (0.175) |
| P for logged |  | (0.012) |  | (0.081) |  | (0.167) |  | (0.065) |
|  |  |  |  |  |  |  |  |  |
| **Prospective (n=2,453)** |  |  |  |  |  |  |  |  |
| **Vitamin B-12** |  |  |  |  |  |  |  |  |
| 1st quartile | 0.00 | [0.00,0.00] | 0.00 | [0.00,0.00] | 0.00 | [0.00,0.00] | 0.00 | [0.00,0.00] |
| 2nd quartile | 0.02 | [-0.08,0.12] | 0.02 | [-0.08,0.13] | 0.04 | [-0.05,0.14] | 0.02 | [-0.08,0.12] |
| 3rd quartile | 0.08 | [-0.02,0.18] | 0.08 | [-0.02,0.18] | 0.09 | [-0.01,0.19] | 0.10^*^ | [0.00,0.20] |
| 4th quartile | 0.07 | [-0.03,0.17] | 0.06 | [-0.04,0.17] | 0.14^**^ | [0.04,0.24] | 0.02 | [-0.08,0.13] |
|  |  |  |  |  |  |  |  |  |
| P for trend |  | (0.093) |  | (0.152) |  | (0.003) |  | (0.357) |
| P for logged |  | (0.035) |  | (0.142) |  | (0.010) |  | (0.651) |

^*^ P ≤ 0.05, ^**^ P ≤ 0.01, ^***^ P ≤ 0.001

Cross-sectional models were adjusted for age, sex, study centre, education, current economic activity, smoking, alcohol, self-reported history of chronic conditions and case-control status.

Prospective models were additionally adjusted for cognitive testing occasion.

P for trend denotes P-value associated with biomarker quartiles modelled as continuous in regression analysis.

P for logged denotes P-value associated with log-transformed biomarker used as independent variable in regression analysis.

# Table A.6 Linear regression results for associations of serum vitamins with standardized cognitive scores excluding participants with extreme biomarker values

|  | **Immediate**  **recall** | | **Delayed**  **recall** | | **Verbal**  **fluency** | | **Letter**  **search** | |
| --- | --- | --- | --- | --- | --- | --- | --- | --- |
|  | **b** | **95% CI** | **b** | **95% CI** | **b** | **95% CI** | **b** | **95% CI** |
| **Cross-sectional (n=3,959)** |  |  |  |  |  |  |  |  |
| **Folate (ng/mL)** |  |  |  |  |  |  |  |  |
| 1st quartile | 0.00 | [0.00,0.00] | 0.00 | [0.00,0.00] | 0.00 | [0.00,0.00] | 0.00 | [0.00,0.00] |
| 2nd quartile | 0.02 | [-0.05,0.10] | 0.05 | [-0.03,0.13] | 0.02 | [-0.05,0.09] | 0.03 | [-0.06,0.11] |
| 3rd quartile | 0.05 | [-0.03,0.12] | 0.04 | [-0.04,0.12] | 0.07 | [-0.00,0.15] | 0.11^*^ | [0.03,0.20] |
| 4th quartile | 0.09^*^ | [0.01,0.17] | 0.05 | [-0.04,0.14] | 0.08^*^ | [0.00,0.16] | 0.12^**^ | [0.03,0.22] |
|  |  |  |  |  |  |  |  |  |
| P for trend |  | (0.020) |  | (0.281) |  | (0.018) |  | (0.001) |
| P for logged |  | (0.010) |  | (0.018) |  | (0.016) |  | (0.000) |
|  |  |  |  |  |  |  |  |  |
| **Prospective (n=2,599)** |  |  |  |  |  |  |  |  |
| **Folate (ng/mL)** |  |  |  |  |  |  |  |  |
| 1st quartile | 0.00 | [0.00,0.00] | 0.00 | [0.00,0.00] | 0.00 | [0.00,0.00] | 0.00 | [0.00,0.00] |
| 2nd quartile | 0.05 | [-0.04,0.15] | 0.01 | [-0.09,0.11] | 0.05 | [-0.04,0.15] | 0.01 | [-0.08,0.11] |
| 3rd quartile | 0.06 | [-0.04,0.16] | -0.01 | [-0.11,0.08] | 0.09 | [-0.00,0.18] | -0.00 | [-0.10,0.09] |
| 4th quartile | 0.11^*^ | [0.01,0.21] | 0.01 | [-0.10,0.11] | 0.15^**^ | [0.05,0.25] | 0.09 | [-0.02,0.19] |
|  |  |  |  |  |  |  |  |  |
| P for trend |  | (0.047) |  | (0.934) |  | (0.002) |  | (0.175) |
| P for logged |  | (0.058) |  | (0.463) |  | (0.009) |  | (0.117) |
|  |  |  |  |  |  |  |  |  |
| **Cross-sectional (n=3,959)** |  |  |  |  |  |  |  |  |
| **Vitamin B-12** |  |  |  |  |  |  |  |  |
| 1st quartile | 0.00 | [0.00,0.00] | 0.00 | [0.00,0.00] | 0.00 | [0.00,0.00] | 0.00 | [0.00,0.00] |
| 2nd quartile | 0.04 | [-0.04,0.11] | 0.06 | [-0.02,0.14] | -0.02 | [-0.09,0.05] | -0.09^*^ | [-0.18,-0.01] |
| 3rd quartile | 0.05 | [-0.03,0.12] | 0.05 | [-0.03,0.13] | -0.00 | [-0.08,0.07] | -0.06 | [-0.14,0.03] |
| 4th quartile | 0.08^*^ | [0.00,0.16] | 0.08 | [-0.01,0.16] | 0.05 | [-0.03,0.12] | -0.05 | [-0.13,0.04] |
|  |  |  |  |  |  |  |  |  |
| P for trend |  | (0.039) |  | (0.102) |  | (0.237) |  | (0.433) |
| P for logged |  | (0.027) |  | (0.109) |  | (0.237) |  | (0.453) |
|  |  |  |  |  |  |  |  |  |
| **Prospective (n=2,603)** |  |  |  |  |  |  |  |  |
| **Vitamin B-12 (pmol/L)** |  |  |  |  |  |  |  |  |
| 1st quartile | 0.00 | [0.00,0.00] | 0.00 | [0.00,0.00] | 0.00 | [0.00,0.00] | 0.00 | [0.00,0.00] |
| 2nd quartile | 0.00 | [-0.09,0.10] | 0.00 | [-0.10,0.10] | 0.01 | [-0.08,0.10] | -0.02 | [-0.12,0.07] |
| 3rd quartile | 0.08 | [-0.01,0.17] | 0.08 | [-0.02,0.18] | 0.06 | [-0.03,0.16] | 0.06 | [-0.04,0.15] |
| 4th quartile | 0.06 | [-0.04,0.16] | 0.05 | [-0.05,0.16] | 0.13^*^ | [0.03,0.23] | -0.01 | [-0.12,0.09] |
|  |  |  |  |  |  |  |  |  |
| P for trend |  | (0.100) |  | (0.138) |  | (0.005) |  | (0.707) |
| P for logged |  | (0.061) |  | (0.161) |  | (0.009) |  | (0.846) |

^*^ P ≤ 0.05, ^**^ P ≤ 0.01, ^***^ P ≤ 0.001

Cross-sectional models were adjusted for age, sex, study centre, education, current economic activity, smoking, alcohol, self-reported history of chronic conditions and case-control status.

Prospective models were additionally adjusted for cognitive testing occasion.

P for trend denotes P-value associated with biomarker quartiles modelled as continuous in regression analysis.

P for logged denotes P-value associated with log-transformed biomarker used as independent variable in regression analysis.

# Table A.7 Linear regression results for associations of serum vitamins with standardized cognitive scores excluding observations with pre-existing CVD or diabetes

|  | **Immediate**  **recall** | | **Delayed**  **recall** | | **Verbal**  **fluency** | | **Letter**  **search** | |
| --- | --- | --- | --- | --- | --- | --- | --- | --- |
|  | **b** | **95% CI** | **b** | **95% CI** | **b** | **95% CI** | **b** | **95% CI** |
| **Cross-sectional (n=3,122)** |  |  |  |  |  |  |  |  |
| **Folate (ng/mL)** |  |  |  |  |  |  |  |  |
| 1st quartile | 0.00 | [0.00,0.00] | 0.00 | [0.00,0.00] | 0.00 | [0.00,0.00] | 0.00 | [0.00,0.00] |
| 2nd quartile | 0.05 | [-0.03,0.14] | 0.08 | [-0.01,0.17] | 0.01 | [-0.08,0.09] | 0.02 | [-0.08,0.12] |
| 3rd quartile | 0.05 | [-0.04,0.13] | 0.06 | [-0.03,0.15] | 0.05 | [-0.03,0.14] | 0.10^*^ | [0.00,0.20] |
| 4th quartile | 0.09^*^ | [0.00,0.18] | 0.09 | [-0.00,0.18] | 0.04 | [-0.05,0.12] | 0.11^*^ | [0.01,0.21] |
|  |  |  |  |  |  |  |  |  |
| P for trend |  | (0.061) |  | (0.104) |  | (0.252) |  | (0.009) |
| P for logged |  | (0.092) |  | (0.016) |  | (0.322) |  | (0.007) |
|  |  |  |  |  |  |  |  |  |
| **Prospective (n=2,139)** |  |  |  |  |  |  |  |  |
| **Folate (ng/mL)** |  |  |  |  |  |  |  |  |
| 1st quartile | 0.00 | [0.00,0.00] | 0.00 | [0.00,0.00] | 0.00 | [0.00,0.00] | 0.00 | [0.00,0.00] |
| 2nd quartile | 0.04 | [-0.07,0.15] | -0.00 | [-0.12,0.11] | 0.03 | [-0.08,0.13] | 0.00 | [-0.10,0.11] |
| 3rd quartile | 0.05 | [-0.06,0.15] | -0.01 | [-0.12,0.10] | 0.07 | [-0.04,0.17] | 0.01 | [-0.10,0.11] |
| 4th quartile | 0.09 | [-0.02,0.20] | 0.00 | [-0.11,0.11] | 0.09 | [-0.02,0.19] | 0.03 | [-0.07,0.14] |
|  |  |  |  |  |  |  |  |  |
| P for trend |  | (0.114) |  | (0.973) |  | (0.072) |  | (0.536) |
| P for logged |  | (0.201) |  | (0.606) |  | (0.119) |  | (0.600) |
|  |  |  |  |  |  |  |  |  |
| **Cross-sectional (n=3,122)** |  |  |  |  |  |  |  |  |
| **Vitamin B-12 (pmol/L)** |  |  |  |  |  |  |  |  |
| 1st quartile | 0.00 | [0.00,0.00] | 0.00 | [0.00,0.00] | 0.00 | [0.00,0.00] | 0.00 | [0.00,0.00] |
| 2nd quartile | 0.09^*^ | [0.00,0.17] | 0.11^*^ | [0.02,0.19] | 0.02 | [-0.06,0.11] | -0.09 | [-0.19,0.00] |
| 3rd quartile | 0.08 | [-0.01,0.16] | 0.09^*^ | [0.00,0.18] | 0.03 | [-0.05,0.12] | -0.04 | [-0.14,0.06] |
| 4th quartile | 0.13^**^ | [0.05,0.22] | 0.12^**^ | [0.03,0.21] | 0.08 | [-0.01,0.16] | -0.01 | [-0.11,0.08] |
|  |  |  |  |  |  |  |  |  |
| P for trend |  | (0.005) |  | (0.014) |  | (0.087) |  | (0.952) |
| P for logged |  | (0.001) |  | (0.006) |  | (0.077) |  | (0.593) |
|  |  |  |  |  |  |  |  |  |
| **Prospective (n=2,139)** |  |  |  |  |  |  |  |  |
| **Vitamin B-12 (pmol/L)** |  |  |  |  |  |  |  |  |
| 1st quartile | 0.00 | [0.00,0.00] | 0.00 | [0.00,0.00] | 0.00 | [0.00,0.00] | 0.00 | [0.00,0.00] |
| 2nd quartile | 0.02 | [-0.08,0.13] | 0.05 | [-0.06,0.16] | 0.03 | [-0.08,0.13] | -0.01 | [-0.12,0.09] |
| 3rd quartile | 0.06 | [-0.04,0.17] | 0.07 | [-0.04,0.18] | 0.06 | [-0.05,0.16] | 0.06 | [-0.04,0.17] |
| 4th quartile | 0.03 | [-0.08,0.13] | 0.04 | [-0.07,0.15] | 0.11^*^ | [0.01,0.22] | -0.01 | [-0.12,0.10] |
|  |  |  |  |  |  |  |  |  |
| P for trend |  | (0.482) |  | (0.430) |  | (0.029) |  | (0.767) |
| P for logged |  | (0.246) |  | (0.281) |  | (0.018) |  | (0.760) |

^*^ P ≤ 0.05, ^**^ P ≤ 0.01, ^***^ P ≤ 0.001

Cross-sectional models were adjusted for age, sex, study centre, education, current economic activity, smoking, alcohol, self-reported history of chronic conditions and case-control status.

Prospective models were additionally adjusted for cognitive testing occasion.

P for trend denotes P-value associated with biomarker quartiles modelled as continuous in regression analysis.

P for logged denotes P-value associated with log-transformed biomarker used as independent variable in regression analysis.
